# Synonymous Site-to-Site Substitution Rate Variation Dramatically Inflates False Positive Rates of Selection Analyses: Ignore at Your Own Peril

**DOI:** 10.1093/molbev/msaa037

**Published:** 2020-02-18

**Authors:** Sadie R Wisotsky, Sergei L Kosakovsky Pond, Stephen D Shank, Spencer V Muse

**Affiliations:** m1 Bioinformatics Research Center, North Carolina State University, Raleigh, NC; m2 Institute for Genomics and Evolutionary Medicine, Temple University, Philadelphia, PA; m3 Department of Statistics, North Carolina State University, Raleigh, NC

**Keywords:** evolutionary model, synonymous rate variation, codon model, episodic selection

## Abstract

Most molecular evolutionary studies of natural selection maintain the decades-old assumption that synonymous substitution rate variation (SRV) across sites within genes occurs at levels that are either nonexistent or negligible. However, numerous studies challenge this assumption from a biological perspective and show that SRV is comparable in magnitude to that of nonsynonymous substitution rate variation. We evaluated the impact of this assumption on methods for inferring selection at the molecular level by incorporating SRV into an existing method (BUSTED) for detecting signatures of episodic diversifying selection in genes. Using simulated data we found that failing to account for even moderate levels of SRV in selection testing is likely to produce intolerably high false positive rates. To evaluate the effect of the SRV assumption on actual inferences we compared results of tests with and without the assumption in an empirical analysis of over 13,000 *Euteleostomi* (bony vertebrate) gene alignments from the Selectome database. This exercise reveals that close to 50% of positive results (i.e., evidence for selection) in empirical analyses disappear when SRV is modeled as part of the statistical analysis and are thus candidates for being false positives. The results from this work add to a growing literature establishing that tests of selection are much more sensitive to certain model assumptions than previously believed.

## Introduction

In 1976, Box (1976) famously wrote “since all models are wrong the scientist must be alert to what is importantly wrong”. As we continue to better understand which aspects of sequence evolution are important to model, existing statistical approaches must be critically reviewed and, as necessary, revised. When originally introduced in 1994 the codon-substitution models (Muse and Gaut 1994; Goldman and Yang 1994) that still form the foundation for most modern tests of natural selection incorporated the then-reasonable assumption that the rate at which synonymous substitutions occur (*dS*) is homogeneous across alignment sites. This assumption makes sense if synonymous substitutions are neutral and the result of underlying constant mutation rate and population parameters (Yang and Nielsen 2008). In contrast, the essential role of modeling the variation in nonsynonymous rates (*dN*) across sites (Nielsen and Yang 1998) and branches (Yang 1998) was appreciated from the outset, because averaging across sites and or branches diminishes statistical power and ignores basic biological realities. In the intervening quarter century two lines of evidence have emerged suggesting that the assumption of *dS* homogeneity is *importantly* wrong. First, models that allow *dS* to vary across alignment sites consistently provide highly significant improvements in goodness-of-fit, for example, in 9/10 cases examined by Kosakovsky Pond and Muse (2005) and in 42% of the almost 8,000 protein groups analyzed by Dimitrieva and Anisimova (2014). Thus, it appears that models most often used in modern statistical analyses of selection fail to capture important aspects of the substitution process: Either variation in *dS* is directly important, or it is confounded with other important unmodeled processes (Jones et al. 2018). Second, dozens of papers now offer examples of natural selection acting on synonymous substitutions. Proposed causes for such selection include secondary RNA structure (Cuevas et al. 2012), codon usage bias (Brandis and Hughes 2016; Kubatko et al. 2016), maintenance of gene function (Eyre-Walker 1996; Lawrie et al. 2013), and effects on a range of mRNA properties: Stability (Chamary and Hurst 2005; Du et al. 2014), alternative splicing (Mueller et al. 2015), and translational efficiency (Shields et al. 1988; Zhou et al. 2010). Many of these examples describe purifying selection on synonymous substitutions, yet instances of positive selection also exist (Resch et al. 2007; Agashe et al. 2016). Furthermore, some synonymous substitutions have strong phenotypic effects: mRNA stability and synthesis of the human dopamine receptor D2 (Duan et al. 2003), driver mutations in human cancers (Supek et al. 2014), and disease association among rare synonymous substitutions in mitochondrial genes (Bhardwaj 2014). A database of deleterious synonymous mutations lists close to 2,000 manually curated human variants (Wen et al. 2016).

We have long promoted the use of models that accommodate site-to-site variation of synonymous substitution rates whenever possible, especially when identifying sites subject to positive or negative selection (Kosakovsky Pond and Frost 2005; Murrell et al. 2012, 2013). We also showed that assuming constant *dS* rates can elevate false-positive rates (FPRs) and lead to loss of power when testing individual sites for selection (Kosakovsky Pond and Frost 2005). Several other groups have also developed models that remove the assumption of synonymous rate homogeneity (e.g., Mayrose et al. 2007; Yang and Nielsen 2008; Zhou et al. 2010; Rubinstein et al. 2011; Zaheri et al. 2014; Kubatko et al. 2016; Davydov et al. 2019). However, when it comes to testing for evidence of natural selection in entire genes, the vast majority of commonly used methods (e.g., those based on the pioneering work of Yang et al. 2002) continues to assume homogeneous synonymous rates. Even our own entry in this domain, BUSTED (Murrell et al. 2015), allows nonsynonymous rates to vary flexibly across branches and sites, yet sets *dS* = 1 as is the current convention.

In this paper, we set out to address the question, “Does the presence of synonymous rate variation (SRV) negatively impact our ability to accurately identify the presence (or absence) of selection acting at the molecular level?*”* To address this question we conducted an extensive simulation study examining the performance of two existing tests of selection, each of which ignores the possibility of SRV. The results show clearly that model misspecification is very costly for standard versions of these methods when SRV is present, with both tests showing unacceptably high FPRs. We also developed a new statistical test, BUSTED[S], by modifying BUSTED to account for the potential presence of SRV (see Materials and Methods). Our tests are rooted in the random-effects modeling framework, where selective pressures vary both across sites and branches, and are well suited for study of pervasive and episodic diversifying selection. The simulations show that this adjustment restores the inflated FPRs to nominal levels. These results raise serious questions about reliability not only for these two particular tests of selection, but of selection tests in general when unaccounted SRV is present. Coupling our simulation study with an empirical analysis of over 13,000 gene alignments we find evidence that roughly half of positive selection findings from the non-SRV methods are likely false positives.

## Results

### A Large-Scale Empirical Screen

We compared the inferences made by using BUSTED[S] to those made by BUSTED in analyses of 13,416 alignments of Eusteleostomes genes extracted from version 6 of the Selectome database (Moretti et al. 2014), which was curated to facilitate the study of positive selection and used previously in Murrell et al. (2015) to benchmark BUSTED. Our goals were to evaluate how frequently SRV was found in real data sets, and to determine how often BUSTED and BUSTED[S] made conflicting inferences (i.e., to explore Box’s “importantly wrong” caution).

For 12,272 of the 13,416 alignments (91.4%), the BUSTED[S] model incorporating SRV was preferred over that of BUSTED using the small sample AIC_*c*_ statistic (Hurvich and Tsai 1989), by a median margin of 112 points (supplementary fig. S1, Supplementary Material online). This result implies that SRV—or processes confounded with it—is the rule rather than the exception. For alignments where BUSTED[S] had the better AIC_*c*_ score, the median coefficient of variation (CV) for synonymous rates was 0.65 with an interquartile range (IQR) of (0.56, 0.78) (supplementary fig. S2, Supplementary Material online). For the remainder of the alignments, the median CV of SRV was 0.48 with IQR (0.29, 0.75). Obviously, all of the data sets that yielded zero estimates for the CV of SRV yield better AIC_*c*_ values for the simpler BUSTED model. For context, the median values for the CVs of nonsynonymous rates estimated by BUSTED[S] were 2.51 and 1.58 for these two groups. Thus, not only is SRV widespread, it tends to be of a magnitude around a quarter of that for nonsynonymous rates—far from negligible.

BUSTED found evidence (likelihood ratio test *P* < .05) of positive selection in 20.4% of the tested data sets, whereas BUSTED[S] did so in 14.8% (table 1). For only 9.3% of the alignments did both methods yield significant results. Importantly, this fact raises the very real possibility that *over one half* (11.1/20.4=54.4%) of all positive results from BUSTED were false positives (see discussion below). A further 5.5% of alignments had positive selection detected only by BUSTED[S]. Cohen’s interrater agreement statistic *κ* (Cohen 1960) was 0.43, indicating only moderate concordance between the methods.

**Table 1.** Selectome Screen for Positive Selection.^a^

	BUSTED[S]		
BUSTED	–	+	Total
–	74% (9,904)	5.5% (742)	79.5% (10,646)
+	11.1% (1,485)	9.3% (1,250)	20.4% (2,735)
Total	85.1% (11,389)	14.8% (1,992)	—

a Percentage (number) of alignments categorized by inferred presence of episodic diversifying positive selection (P≤0.05) using BUSTED (rows) and BUSTED[S] (columns). + denotes selection inferred, – indicates no selection found.

**Fig. 1.**
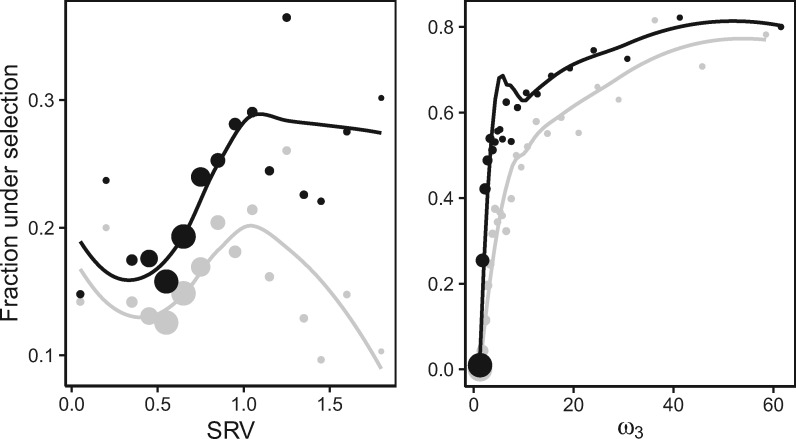
Fraction of Selectome alignments inferred to be under selection. Plotted points indicate the fraction of Selectome data sets inferred to have positive selection (P≤0.05). Each disc represents the average for at least 100 alignments with the size representing the relative number of alignments in each bin for BUSTED (black) and BUSTED[S] (gray). Smoothing curves are weighted Loess polynomials. SRV—coefficient of variation for synonymous rates, as inferred by BUSTED[S], *ω*_3_—the maximum likelihood estimate of *ω*_3_.

Further investigation reveals that the probability of an alignment yielding a significant selection result is strongly impacted by the magnitude of SRV in that data set, and that this effect is more pronounced in BUSTED (fig. 1). For data sets with a synonymous rate CV near zero the rejection rates for the two methods are virtually identical, as one would expect (and as shown below, this behavior is also supported by simulation). As the amount of SRV increases, though, the discrepancy between the rejection rates of BUSTED and BUSTED[S] grows, offering evidence that BUSTED may be “interpreting” variation in synonymous rates as a (potentially false) signal for positive selection. As levels of SRV continue to increase, both methods seemingly lose power, possibly due to a saturation effect, and show patterns similar to each other for increasing codon and sequence lengths (supplementary fig. S4, Supplementary Material online). We found no evidence that the synonymous rate CV is a simple correlate of another data feature (e.g., sequence length, tree length, intensity of selection, etc., see supplementary fig. S5, Supplementary Material online).

**Fig. 2.**
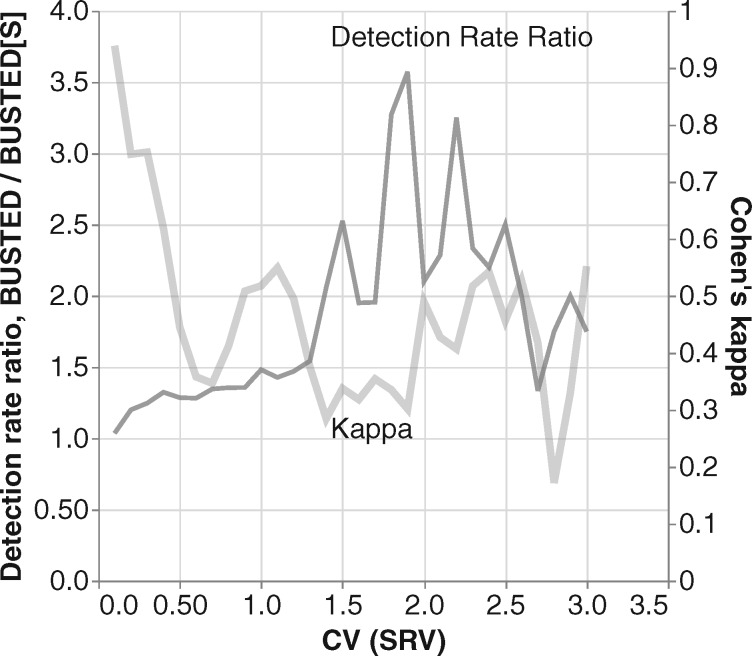
Comparison of method results on Selectome data as a function of the level of SRV. The two plots show relationships between the level of SRV (*x*-axis) and either the relative selection detection rates of BUSTED and BUSTED[S] (left *y*-axis, black plot) or Cohen’s measure of method concordance (right *y*-axis, gray plot). The plots are sliding window analyses using a window size of 0.2 and a step size of 0.1. Windows with <10 points are not plotted to reduce noise.

These analyses reveal that the magnitude of SRV is a major factor influencing both the relative detection rates (i.e., power) of these two methods and the level of agreement between them (fig. 2). For data sets with minimal SRV, (i.e., estimated CV(SRV)<0.1), BUSTED and BUSTED[S] have essentially identical detection rates, and the value of Cohen’s *κ* near 0.9 indicates near perfect agreement. Good agreement (κ≥0.8) is maintained up to CV(SRV)≈0.4, but agreement quickly plummets. By the time CV(SRV)=0.5 agreement has reached κ≈0.45 and BUSTED begins to detect selection in 25–30% more data sets than BUSTED[S]. As CV(SRV) passes 1.0 BUSTED rejects 50% more often, and this detection ratio climbs quickly as SRV increases in magnitude, topping out at over 300% when CV(SRV) nears 2.0. As we show using simulations in the next section, CV(SRV)≈0.5 appears to be the critical threshold at which BUSTED develops very high levels of false positives.

This pattern of method behavior is consistent with the following interpretation. For data sets where there is no or little synonymous rate variation, nearly identical results are obtained regardless of whether or not SRV is modeled (this also implies that BUSTED[S] does not lose much power relative to BUSTED). However, for data sets where CV(SRV) is sufficiently high, failing to model SRV drives BUSTED’s rate of detection far above that of BUSTED[S]—as much as 2–3-fold higher. Based on evidence from the simulations described below, we argue that this excess is likely the result of BUSTED false positives that could be avoided by incorporating SRV into the statistical testing procedure.

### Analysis of Reference Data Sets

We performed an in-depth analysis of 11 additional data sets that have been previously featured in studies of SRV and positive selection screening:

- Nine of the ten data sets used in the original SRV work of Kosakovsky Pond and Muse (2005) (we replaced the Influenza A virus alignment from this reference with one from Chen and Sun 2011, see below)
- The Rhodopsin gene alignment from Yokoyama et al. (2008)—a source of a vigorous debate in the context of positive selection screening (Nozawa et al. 2009; Yang et al. 2009)
- The Influenza A virus HA alignment from Chen and Sun (2011); this particular alignment was analyzed by the original authors to showcase the sensitivity of methods for detecting pervasive positive selection to sampling and variation in selective pressure.

In these 11 data sets, AIC_*c*_ scores universally favor BUSTED[S] by sizable margins (median AIC_*c*_ difference of 160.49 points, see table 2), and the synonymous rate CVs are all at least 0.5, once again supporting the claim of widespread SRV. Seven of eleven data sets were found to be under episodic diversifying selection by both methods (P≤0.05), and neither method detected selection in the remaining four. However, the inclusion of SRV in the statistical analysis can significantly impact parameter estimates. For example, the estimate of *ω*_3_, the intensity of positive selection for the positively selected class of branches/sites, is lowered by a factor of three in the rhodopsin data set and by a factor of two in the *β*-globin data set. Similarly, the fraction (*p*_3_) of sites subject to selection is halved in the Camelid VHH data set. For HIV-1 *vif*, BUSTED[S] characterizes selection as being very strong (an effectively infinite *ω*_3_ estimate) but present only at a very small proportion of branches/sites, whereas BUSTED suggests a much more subdued estimate of *ω*_3_ at a fraction of branches/sites several orders of magnitude greater. The inferred distributions of synonymous substitution rates ran the gamut of distributional composition. In COXI, 95% of the sites appear to evolve at the mean rate, 2% at a very low rate, and 3% at a very high rate (more than 100× higher than the low rate). In Camelid VHH, the sites are binned into low, medium, and high rates (10× higher that the low rate) with roughly equal proportions.

**Table 2.** Analysis of Reference Data Sets.^a^

			BUSTED				BUSTED[S]						Sites			
Gene	S	N	*P* Value	CV(ω)	*ω*_3_	*p*_3_	*P* Value	CV(ω)	CV(α)	*ω*_3_	*p*_3_	*α* Distribution	ΔAIC_*c*_	++	+−	−+
*β*-Globin	17	144	$<10^{-4}$	4.66	20.54	2.7%	$<10^{-4}$	3.96	1.46	9.60	3.4%	0.42 (53%), 1.3 (44%), 6.5 (3%)	37.13	4	2	0
Flavivirus NS5	18	342	0.42	4.17	1.14	4.4%	0.49	4.60	1.71	1.11	2%	0.12 (19%), 0.53 (72%), 6.2 (9%)	259.28	0	0	0
Primate COXI	21	510	0.5	5.60	1.00	2.5%	0.5	6.32	2.35	1.00	1.1%	0.04 (2%), 0.58 (95%), 13.5 (3%)	105.59	0	0	0
Drosophila *adh*	23	254	0.0003	4.73	4.78	2.6%	0.0016	4.62	0.59	4.26	2.4%	0.53 (40%), 1.0 (52%), 3.0 (8%)	18.02	1	0	0
Encephalitis *env*	23	500	0.5	0.0	1.00	0%	0.5	0.0	0.67	1.14	0%	0.41 (31%), 1.1 (66%), 4.6 (3%)	46.32	0	0	0
Sperm lysin	25	134	$<10^{-4}$	2.45	17.13	10%	$<10^{-4}$	2.39	0.87	17.38	7.6%	0.21 (38%), 1.1 (46%), 2.5 (16%)	160.49	23	2	0
HIV-1 *vif*	29	192	0.0002	1.68	3.14	26%	0.025	18.28	1.06	988.84	0.05%	0.30 (54%), 1.2 (34%), 3.6 (12%)	188.31	0	1	0
Hepatitis D virus antigen	33	196	$<10^{-4}$	3.81	16.42	2.9%	$<10^{-4}$	3.58	0.92	16.61	1.9%	0.05 (22%), 0.78 (58%), 2.7 (20%)	273.38	8	2	0
Vertebrate Rhodopsin	38	330	$<10^{-4}$	6.78	20.76	1%	$<10^{-4}$	5.57	1.42	7.06	1.0%	0.36 (57%), 1.1 (38%), 8.2 (5%)	473.77	5	3	0
Influenza A virus HA	86	329	0.5	2.14	1.00	27%	0.095	1.11	0.85	2.10	18%	0.49 (62%), 1.3 (29%), 3.4 (9%)	130.9	0	0	0
Camelid VHH	212	96	$<10^{-4}$	3.22	28.51	3.9%	$<10^{-4}$	3.05	0.84	26.87	1.9%	0.24 (33%), 0.85 (45%), 2.5 (22%)	1,436.46	26	11	1

a Results from the reanalysis of the data sets used in Kosakovsky Pond and Muse (2005), Yokoyama et al. (2008), and Chen and Sun (2011), arranged by sequence count. We ran selection tests with three nonsynonymous and three synonymous rate categories (for BUSTED[S]). Column headings are as follows: *S*, number of sequences; *N*, number of codons; CV(ω), the coefficient of variation (CV) for the inferred distribution of *ω* ratios; *ω*_3_, the maximum likelihood estimate (MLE) of the strength of selection; *p*_3_, the MLE of the proportion of sites under selection (proportion of sites in the *ω*_3_ category); CV(α), the CV for the inferred distribution of synonymous rates; ΔAIC_*c*_, the difference between AIC_*c*_ values of BUSTED and BUSTED[S]. The *α* distribution columns list the estimated values of the 3 *α* categories along with their estimated frequencies. The Sites columns count the number of alignments where at least one method called a site selected (using evidence ratio of at least 5): ++ both methods yes; +− BUSTED yes, BUSTED[S] no; −+ BUSTED no, BUSTED[S] yes.

Although BUSTED[S] is not optimized for site-wise selection analysis (we instead recommend the MEME procedure of Murrell et al. 2012), site-level evidence ratios (ER) or factor loadings provide a quantitative indication of which sites may be contributing to the signal for positive selection, and what types of sites have discordant rate preferences between BUSTED and BUSTED[S] analyses. As detailed in Murrell et al. (2015), ER are simply likelihood ratios of two models evaluated on the data from a specific site: The unconstrained model (the selection intensity *ω*_3_ is estimated) and the constrained model ($\omega_{3}=1$). A high ER at a site implies that the data at that site have a higher relative likelihood when positive selection is permitted. Table 2 highlights the subsets of sites where ER classification agreed or disagreed between methods. For instance, for the Camelid VHH data set, 26 sites were classified as preferring the positive selection regime (ER > 5) for both methods, 11 sites had ER≥5 for BUSTED but ER≤1 for BUSTED[S], and one site had ER≥5 for BUSTED[S] but ER≤1 for BUSTED.

**Fig. 3.**
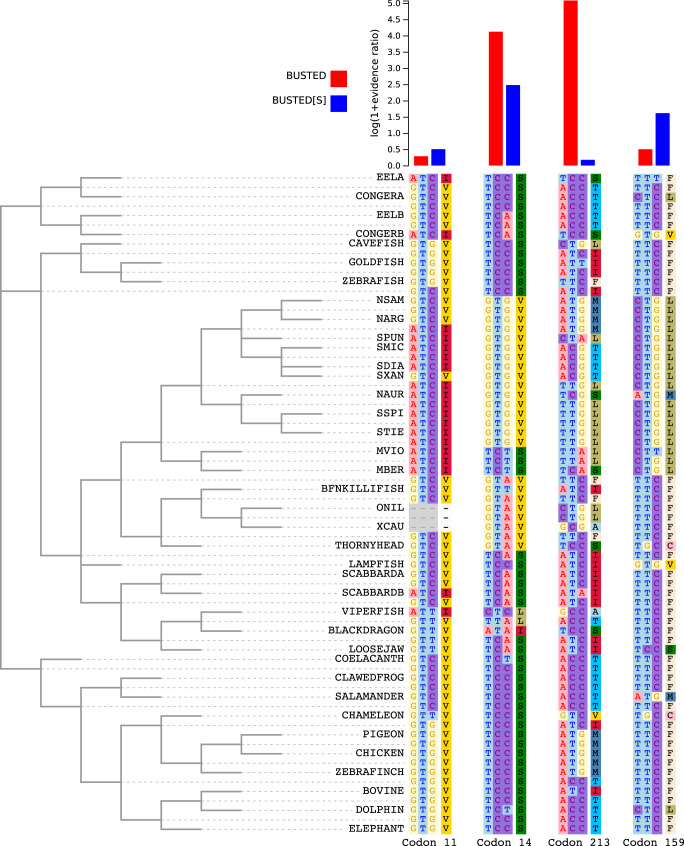
Sample sites illustrating tendencies of BUSTED and BUSTED[S]. Phylogenetic tree for the Rhodopsin gene alignment with the evolutionary histories of four codons representing informative cases of agreement and disagreement between BUSTED[S] and BUSTED. See text for details.

Figure 3 shows four sites from the rhodopsin alignment that provide insight into how selection status and substitution rates are classified by BUSTED and BUSTED[S]. Codon 11 has four synonymous and seven nonsynonymous substitutions inferred via joint likelihood reconstruction as implemented in SLAC (Kosakovsky Pond and Frost 2005) and a posterior estimate of mean site-specific synonymous rate (denoted by *α*), $\hat{\alpha }=1.16$. This site is classified by both methods as having low support for positive selection. Codon 14 ($\hat{\alpha }=5.1$) is flagged by both methods as showing evidence for episodic selection (it reveals a cluster of substitutions). Codon 213 shows very strong evidence for positive selection when using BUSTED, but BUSTED[S] provides virtually no such evidence; the very high estimate of $\hat{\alpha }=6.25$ suggests that the codon may be hypervariable. Lastly, codon 159 with 4 synonymous and 12 nonsynonymous inferred substitutions has stronger evidence for selection under BUSTED[S] than BUSTED; its estimated $\hat{\alpha }=0.75$ is somewhat less than the alignment average, thereby boosting the *dN*/*dS* ratio.

To summarize, even when BUSTED and BUSTED[S] agree on the “big picture” question—is the gene under selection?—key parameter estimates and downstream inferences about sites contributing to the signal of selection can differ rather markedly between the two methods. Consequently, any secondary analyses that depend on parameter estimates or site-level inferences will likely be impacted by the decision to model or ignore SRV.

### Simulation Study

Since it is logically impossible to unambiguously evaluate the rates of false positive and false negative results using empirical data alone, we carried out an extensive simulation study to evaluate the statistical properties of BUSTED and BUSTED[S] for varying levels of SRV. (For technical simulation details see supplementary materials, Supplementary Material online and supplementary figs. S9 and S10, Supplementary Material online.) We chose sequence lengths ranging from 100 codons (typical protein-coding gene) to 5,000 codons (eliminate the effects of sampling). We report results from a model tree with 31 sequences, and very similar results arising from a 16-sequence model tree are found in supplementary figure S7, Supplementary Material online. These trees were chosen based on the typical sizes of the Selectome alignments used in our empirical study. To help us understand the effects of SRV on the methods’ power we simulated data using empirically derived ranges for the level of SRV and the intensity of selection (*ω*_3_).

#### Type I Error Rates

**Fig. 4.**
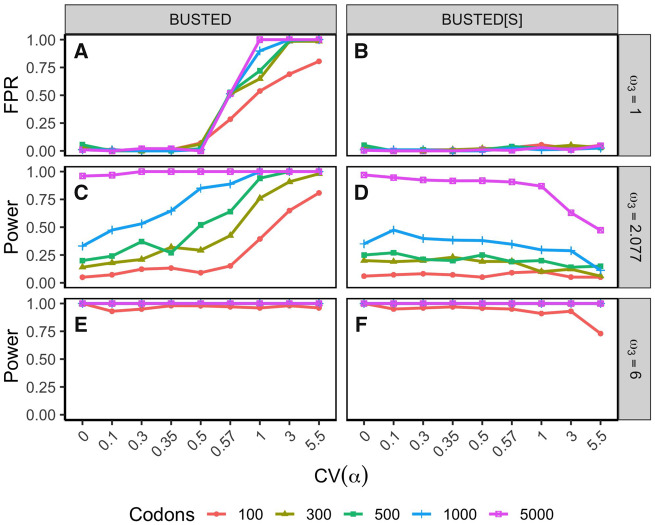
Method performance with simulated data of 31 sequences as a function of the amount of synonymous rate variation (CV(α)) and the strength of selection (*ω*_3_). For each combination of CV(α) and *ω*_3_, we simulated 100 alignments and applied BUSTED and BUSTED[S]. $\omega_{3}=1$—neutral evolution (null) (*A*, *B*), $\omega_{3}=2.077$—moderate selection (*C*, *D*), and $\omega_{3}=6$—strong selection (*E*, *F*). Data were simulated with 3% of branch-site combinations under selection. Plotted points are the frequencies of simulated data sets where selection was inferred (i.e., False Positive Rate [FPR] when $\omega_{3}=1$, Power when $\omega_{3}>1$). Line colors indicate sequence length. Note that the horizontal axis is not linear in scale.

When data were simulated with no positive selection and no SRV, both BUSTED and BUSTED[S] showed Type I errors at or beneath the nominal levels: supplementary figure S6, Supplementary Material online reveals frequencies consistent with the uniform distribution of *P*-values predicted by theory. However, we need to understand the Type I error rates when SRV is present. Panels A and B of figure 4 describe results from data simulated without selection ($\omega_{3}=1$). For these data BUSTED[S] maintained the proper nominal Type I error rate (0.05) regardless of the extent of SRV or the length of the sequence. In stark contrast, once the CV of SRV exceeded 0.5 FPRs for BUSTED quickly rose to around 50% and approached 100% when the CV exceeded 1. Recall that well over half of the Selectome data sets had CV(SRV) >0.5, and that this was the point where BUSTED and BUSTED[S] began to substantially deviate in their inferences on those data sets (fig. 2). This catastrophic loss of Type I error control for levels of SRV common in real data is clearly undesirable.

**Fig. 5.**
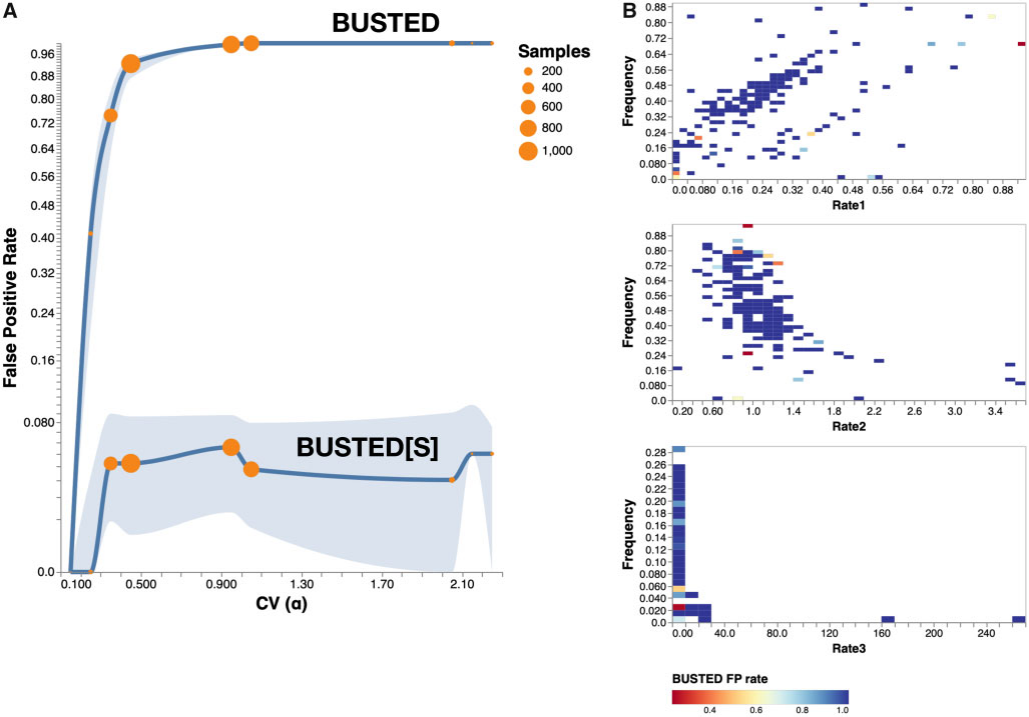
Method performance on null simulations with varied distributions of rates. (*A*) FPRs of BUSTED and BUSTED[S] as a function of simulated CV(α) on neutrally evolving data; the distributions of rates used for simulations were varied and derived from a large empirical data set of avian genes analyzed by Shultz and Sackton (2019). The solid curve is the rate for nominal *P *=* *0.05, and the shaded areas delimit the corresponding values for *P *=* *0.01 (lower bound) and *P *=* *0.1 (upper bound). The number of simulations used to estimate rates for each bin of CV(α) is reflected in the size of the circle. Note the nonlinear scale on the *y*-axis. (*B*) The rate at which BUSTED makes false positive errors (at nominal *P *=* *0.05), as a function of the *α* values used in the simulations. The plot is restricted to data sets where CV(α)>0.4, which is the value where the catastrophic loss of false positive rate control begins. Because the distributions were drawn from empirical alignments, they reflect what is encountered in biological data but do not fill the parameter space completely; because the *α* distribution must have unit mean, some combination of rates and frequencies are not feasible (e.g., the maximum frequency of $\alpha_{3}>1$ cannot exceed $1/\alpha_{3}$).

There are many distributional, substitution rate, tree shape, and base frequency parameters that could influence the statistical behavior of the methods. Rate distributions might be symmetric or skewed; discrete rate distributions with the same CV might have different allocations of rates to classes (e.g., a small frequency for an extreme rate value, or larger frequencies for rates closer to the mean); trees might or might not be balanced, etc. Designing a simulation experiment to explore the full range of possible combinations would be a massive undertaking. However, in an effort to provide some understanding of how Type I error rates respond to perturbations of these factors, we took a large collection of empirical alignments that represent a subset of the potential parameter space and simulated data sets using values estimated from each of those alignments. More specifically, we chose a collection of avian protein-coding gene alignments previously analyzed for evidence of selection by Shultz and Sackton (2019), estimated maximum likelihood trees for each alignment using RAxML-NG (Kozlov et al. 2019) with default settings, and inferred rate parameters using BUSTED[S] (since it allows estimation of SRV). These estimated values were then used to parametrically simulate long (5,000 codons) sequence alignments under strict neutrality, and BUSTED and BUSTED[S] were applied to each simulated data set. We used equal base frequencies and set *κ *= 2 in the HKY85 nucleotide model component (see Materials and Methods) to isolate differences between simulation replicates to the tree topology and the rate distribution. The simulations included 3,278 data sets that cover a wide range of distributional representations of *α* (supplementary fig. S3, Supplementary Material online), whereas our initial simulations examined only a single rate distribution for a given value of CV. A similar pattern of statistical behavior emerges from this set of simulations (fig. 5*A*): BUSTED has a rate of false positives that grows rapidly as a function of CV(α) and reaches 100% for CV(α)>0.5, whereas BUSTED[S] maintains roughly nominal error rates for the entire range of CV(α). FPR behavior is not notably influenced by the higher moments of the CV(α) distribution, or by the fraction or extent of “extreme” rates (fig. 5*B*).

As a further confirmation of the generality of this behavior—this time with a “sites model” rather than a “branch-site model”—we also analyzed our simulated data using the M1a-M2a procedure of Wong et al. (2004) described in Materials and Methods. While nominal FPRs are observed when no SRV is present, the Type I error rate pathology is even more extreme for M1a-M2a than it was for BUSTED: 100% FPRs are reached with even lower levels of SRV (supplementary fig. S8, Supplementary Material online). A recently published work (Davydov et al. 2019) showed high FPRs for several positive selection tests in the presence of SRV (including one method that was essentially a different parameterization of BUSTED), further supporting this conclusion. More generally, Davydov et al. (2019) showed that a variety of modeling decisions can have substantial effects on FPRs for selection tests. These methods do not seem to have the robustness that has been widely suspected.

#### Power

Figure 4*C–F* allows us to study the impacts of SRV on the statistical power of the two methods. For data simulated under strong selection ($\omega_{3}=6$), both methods have power near 100%. At a more moderate level of selection ($\omega_{3}=2.077$) shorter sequences result in lower power across all levels of SRV. Although BUSTED has considerable power in this setting (panel C), comparison of its behavior to that in panel A ($\omega_{3}=1$) reveals that much of the apparent power is likely an artifact arising from the presence of SRV. In other words, if we “subtract out” BUSTED’s false positives from panel A, the power curves in panel C would look very much like those of BUSTED[S] in panel D. BUSTED[S] does lose power when high levels of SRV are present as seen in panel D, echoing the behavior seen in the Selectome analysis. However, BUSTED[S] does *not* suffer from power loss compared with BUSTED for low to moderate levels of SRV, where Type I errors of the methods are comparable.

The impact of this behavior is not merely theoretical, it has already been demonstrated to alter inferences from published analyses. Examples for site-level rate inference were highlighted in (Kosakovsky Pond and Muse 2005), and we showed above an example of a “hypervariable” site in the Rhodopsin gene alignment at codon 213 (fig. 3). Discordantly classified sites are also relatively frequent in other example data sets (table 2). Intuitively, a site where both synonymous and nonsynonymous rates are high, but with ω≤1, may be placed in the high *ω* category by methods such as BUSTED or M1a-M2a that cannot accommodate variable synonymous rates. Recent elegant work by Jones et al. (2018) on what they call phenomenological load on model parameters revealed the tendency for available model parameters to absorb unmodeled sources of variation when the model is misspecified. In the current case, these tests of selection seemingly absorb some of the synonymous rate variation into elevated values of *ω* parameters.

Jones et al. (2018) further examined whether or not SRV itself could be attributed to phenomenological load, that is, is detection of SRV merely an artifact of another process that the model ignores? Encouragingly, these authors found that, according to their framework, SRV appeared to be a genuine effect in biological data sets examined. Our work supports their finding, showing that the estimated magnitude of SRV is not obviously explained by simple biological factors.

## Discussion

That synonymous substitutions are not fully neutral is no longer a matter of debate. The combination of empirical and simulation results above demonstrates that the necessity of modeling synonymous rate variation in computational screens for natural selection should also be a settled issue. We developed a new method (BUSTED[S]) by adding the ability to account for SRV to an existing method for detecting gene-level episodic selection (BUSTED), and used it to screen a large collection of vertebrate gene alignments for evidence of selection. This screen revealed that over 50% of positive results found by the SRV-agnostic BUSTED disappear when SRV is explicitly modeled. There are two obvious explanations for this discrepancy: Either BUSTED suffers from a high rate of false positive in the presence of SRV, or BUSTED[S] suffers from low power. Extensive simulation studies of these methods showed that the presence of sufficient levels of SRV quickly caused BUSTED to have FPRs near 100%, a result that invokes the memory of maximum parsimony’s “positively misleading” behavior when the molecular clock assumption is violated (Felsenstein 1978). While BUSTED[S] did show somewhat reduced power compared with BUSTED, the reduction was relatively small unless exceptionally high levels of SRV were present. A large majority of the Selectome data sets had CV(SRV) values <1.5, well below the point where BUSTED[S] begins to lose power. In contrast, the majority of Selectome data sets also had levels of SRV above 0.5, the point at which BUSTED’s FPRs sharply increase.

This combination of empirical and simulation results strongly suggests that a large fraction of significant BUSTED tests in the Selectome analyses are, in fact, false positives. The finding that M1a-M2a also shows potentially catastrophic Type I error rate problems, coupled with the prevalence of SRV in real data, raises the more general concern that many reported instances of positive selection in the literature might actually be false positives. Consequently there is no compelling reason *not* to model SRV when conducting selection analyses: If there is not too much SRV in the data, we obtain results that are nearly identical to traditional models, and if there is enough (a reasonable a priori assumption based on empirical studies), then the cost of ignoring SRV is an unacceptably high rate of false positives. Because the addition of SRV to standard codon models is not unduly computationally taxing (∼3−5× longer run times in BUSTED[S], for example), we strongly encourage anyone interested in studies of gene-wide selection to switch to SRV-enabled models.

The empirical and simulation work in this paper adds to a growing body of literature strongly suggesting that the models underlying these methods—not only BUSTED, but almost certainly any positive selection method that assumes the absence of SRV—are *importantly* wrong and need to be revised.

## Materials and Methods

### Statistical Methodology

We adapted the existing BUSTED test of positive selection (Murrell et al. 2015) to account for the presence of SRV and call the new method BUSTED[S]. To explore the generality of our findings about FPRs in the presence of SRV we also investigated a second existing test of selection, the M1a versus M2a comparison from Wong et al. (2004), modified slightly to employ MG94 substitution models.

BUSTED[S] is a straightforward extension of BUSTED (Murrell et al. 2015). The nucleotide substitution process is modeled using the standard finite state continuous time Markov process approach of Muse and Gaut (1994), with entries of the instantaneous rate matrix *Q* corresponding to substitutions between sense codons *i* and *j* denoted as

$$q_{ij}=\{\begin{array}{cc} \alpha^{s}\theta_{ij}\pi_{j}^{p} & 1-\text{step}\;\text{synonymous}\;\text{change},\\ \alpha^{s}\omega^{bs}\theta_{ij}\pi_{j}^{p} & 1-\text{step}\;\text{nonsynonymous}\;\text{change},\\ 0 & \text{otherwise}. \end{array}$$

The *θ_ij_* (with $\theta_{ij}=\theta_{ji}$) are parameters governing nucleotide substitution biases. For example, $\theta_{\text{ACT},\text{AGT}}=\theta_{\text{CG}}$ and because we incorporate the standard nucleotide GTR model there are five identifiable *θ_ij_* parameters: $\theta_{\text{AC}},\;\theta_{\text{AT}},\;\theta_{\text{CG}},\;\theta_{\text{CT}}$, and $\theta_{\text{GT}}$, with $\theta_{\text{AG}}\equiv 1$. The position-specific equilibrium frequency of the target nucleotide of a substitution is $\pi_{j}^{p}$; for example, it is $\pi_{G}^{2}$ for the second-position change associated with $q_{\text{ACT},\text{AGT}}$. The $\pi_{j}^{p}$ and the stationary frequencies of codons under this model are estimated using the CF3 × 4 procedure (Kosakovsky Pond et al. 2010), adding nine parameters to the model. The ratio of nonsynonymous to synonymous substitution rates for site *s* along branch *b* is *ω^bs^*, and this ratio is modeled using a 3-bin general discrete distribution (GDD) with five estimated hyperparameters: $0\leq \omega_{1}\leq \omega_{2}\leq 1\leq \omega_{3},\;p_{1}=P(\omega^{bs}=\omega_{1})$, and $p_{2}=P(\omega^{bs}=\omega_{2})$. The procedure for efficient computation of the phylogenetic likelihood function for these models was described in Kosakovsky Pond et al. (2011). The quantity *α^s^* is a site-specific synonymous substitution rate (no branch-to-branch variation is modeled) drawn from a separate 3-bin GDD. The mean of this distribution is constrained equal to one to maintain statistical identifiability, resulting in four estimated hyperparameters: $0\leq c\alpha_{1}<\alpha_{2}=c\leq c\alpha_{3},\;f_{1}=P(\alpha^{s}=\alpha_{1})$, and $f_{2}=P(\alpha^{s}=\alpha_{2})$, with *c* chosen to ensure that $E\{\alpha^{s}\}=1$. Typical implementations, including ours, allow the number of *α* and *ω* rate categories to be separately adjusted by the user, for example, to minimize AIC_*c*_ or to optimize some other measure of model fit. The default setting of three categories generally provides a good balance between fit and performance when using this GDD approach for modeling. Our HyPhy implementation of BUSTED[S] will warn the user if there is evidence of model overfitting, such as the appearance of rate categories with very similar estimated rate values or very low frequencies.

The BUSTED[S] procedure for identifying positive selection is the likelihood ratio test comparing the full model described above to the constrained model formed when *ω*_3_ is set equal to 1 (i.e., no positively selected sites). Critical values of the test are derived from a 50:50 mixture distribution of $\chi_{0}^{2}$ and $\chi_{2}^{2}$. Note that this asymptotic statistic differs from the 3-component mixture used by Murrell et al. (2015); the simulation studies performed in the current study suggest that this less conservative mixture is sufficient to maintain nominal Type I errors. Both BUSTED[S] and BUSTED analyses in the current work use the same 50:50 mixture test statistic. BUSTED[S] reduces to BUSTED by setting $\alpha^{s}=1$, that is, by placing all the mass of the synonymous rate heterogeneity distribution at *α *= 1. The method is implemented as a part of HyPhy (version 2.5.1 or later). BUSTED[S] is available for free public use on the Datamonkey webserver (Weaver et al. 2018) at https://www.datamonkey.org/BUSTED (last accessed February 24, 2020).

### Selectome Data and Alignments

Data and alignments for the empirical analyses come directly from version 6 of the Selectome database (Moretti et al. 2014). NEXUS-format files used for analysis here can be downloaded from data.hyphy.org/web/busteds/ (last accessed February 24, 2020).

### Simulation Data

Simulated data sets can be downloaded from data.hyphy.org/web/busteds/ (last accessed February 24, 2020). See supplementary figures S9 and S10, Supplementary Material online for model tree information. Additional information is present in the README.md file, including details of how to generate alignments under the BUSTED[S] models.

## Supplementary Material

msaa037_Supplementary_Data
