# Supplementary figures and images for: Synonymous Site-to-Site Substitution Rate Variation Dramatically Inflates False Positive Rates of Selection Analyses: Ignore at Your Own Peril
Source: Mol Biol Evol. 2020 Feb 18;37(8):2430–9. doi: 10.1093/molbev/msaa037 (PMC7403620; doi:10.1093/molbev/msaa037)

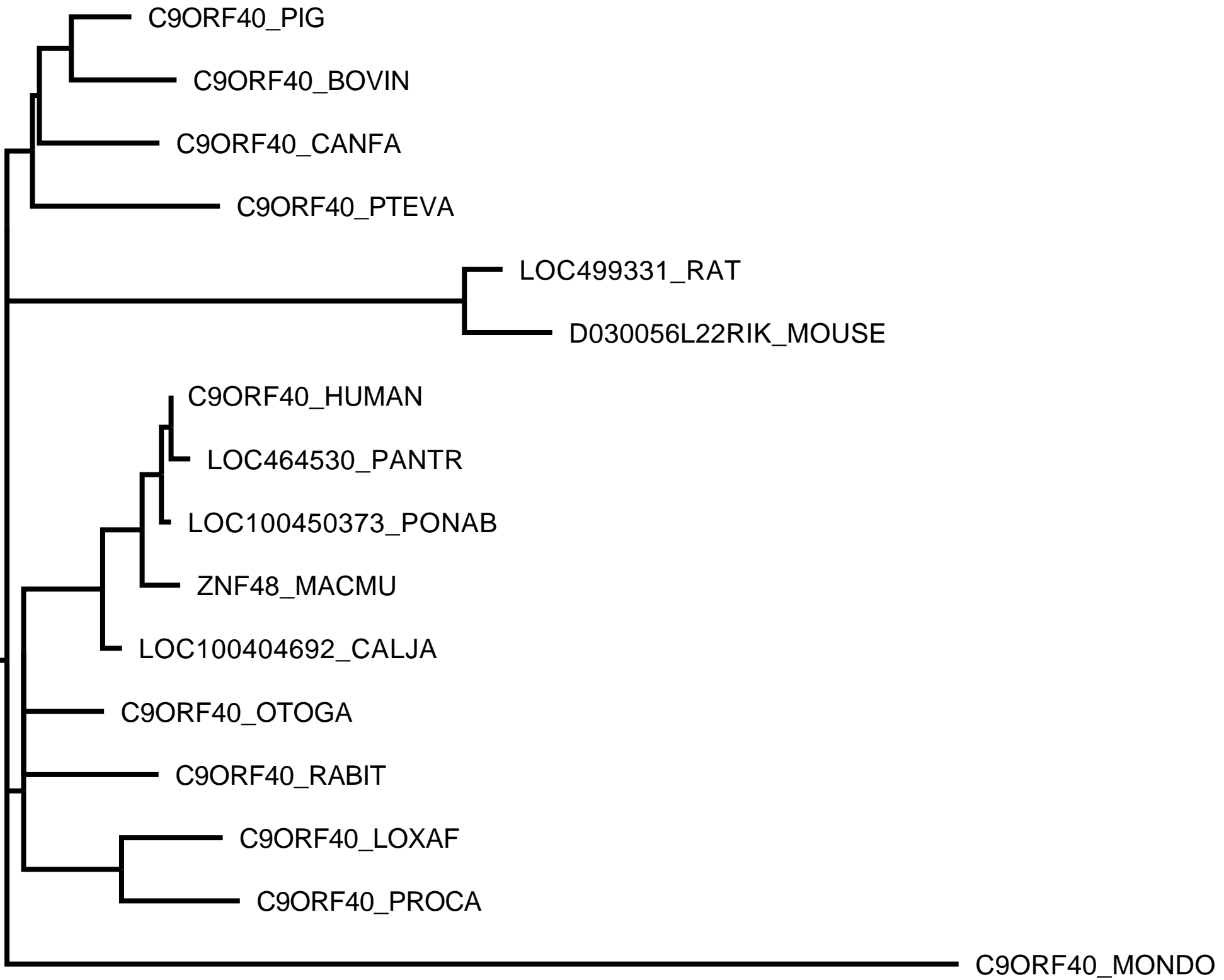

0.04

Supplement: msaa037_Supplementary_Data [file msaa037_supplementary_data.zip › msaa037-suppl-data/S10_Model_Tree_16_seq.pdf]

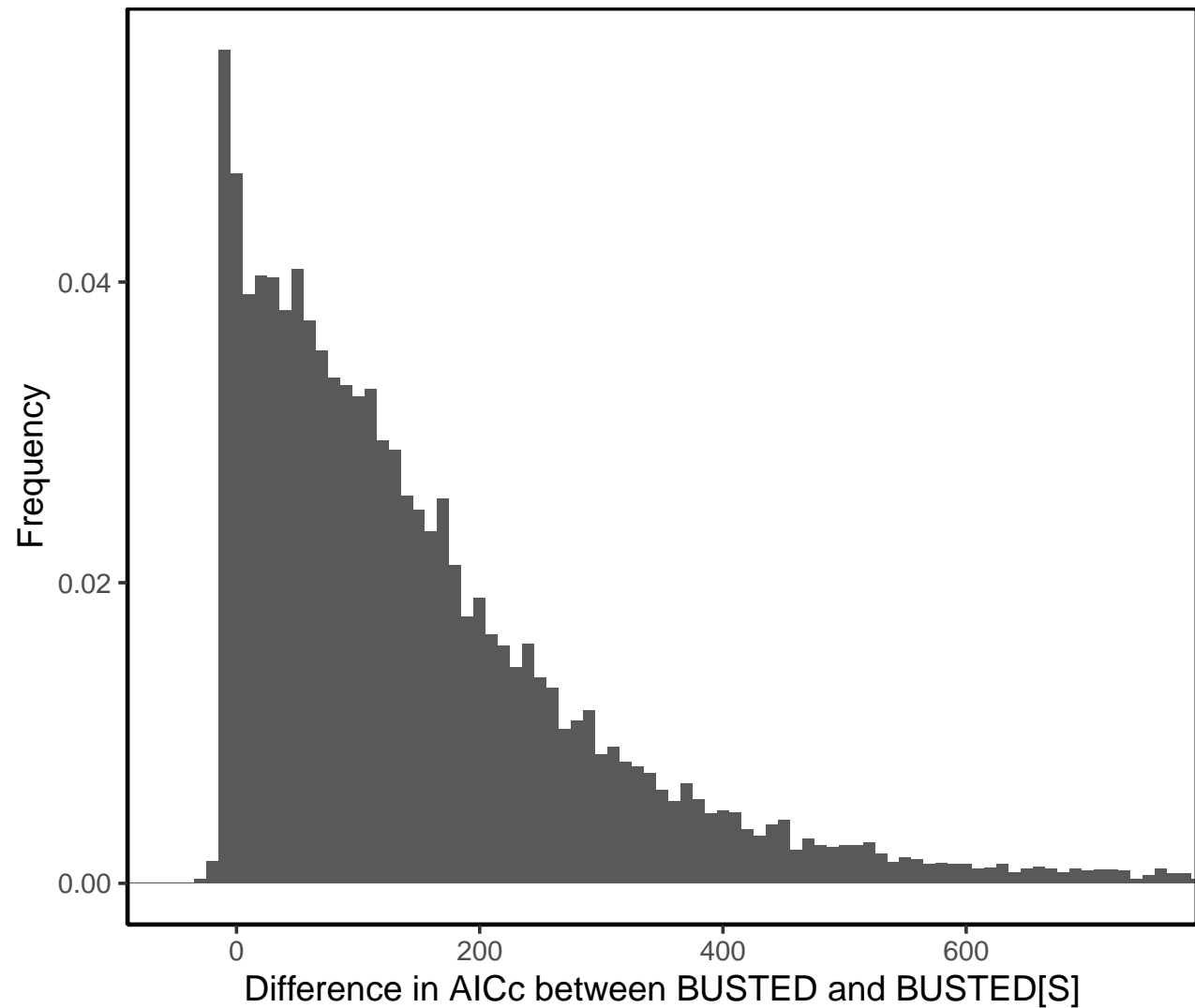

Supplement: msaa037_Supplementary_Data [file msaa037_supplementary_data.zip › msaa037-suppl-data/S1_AICc_Hist.pdf]

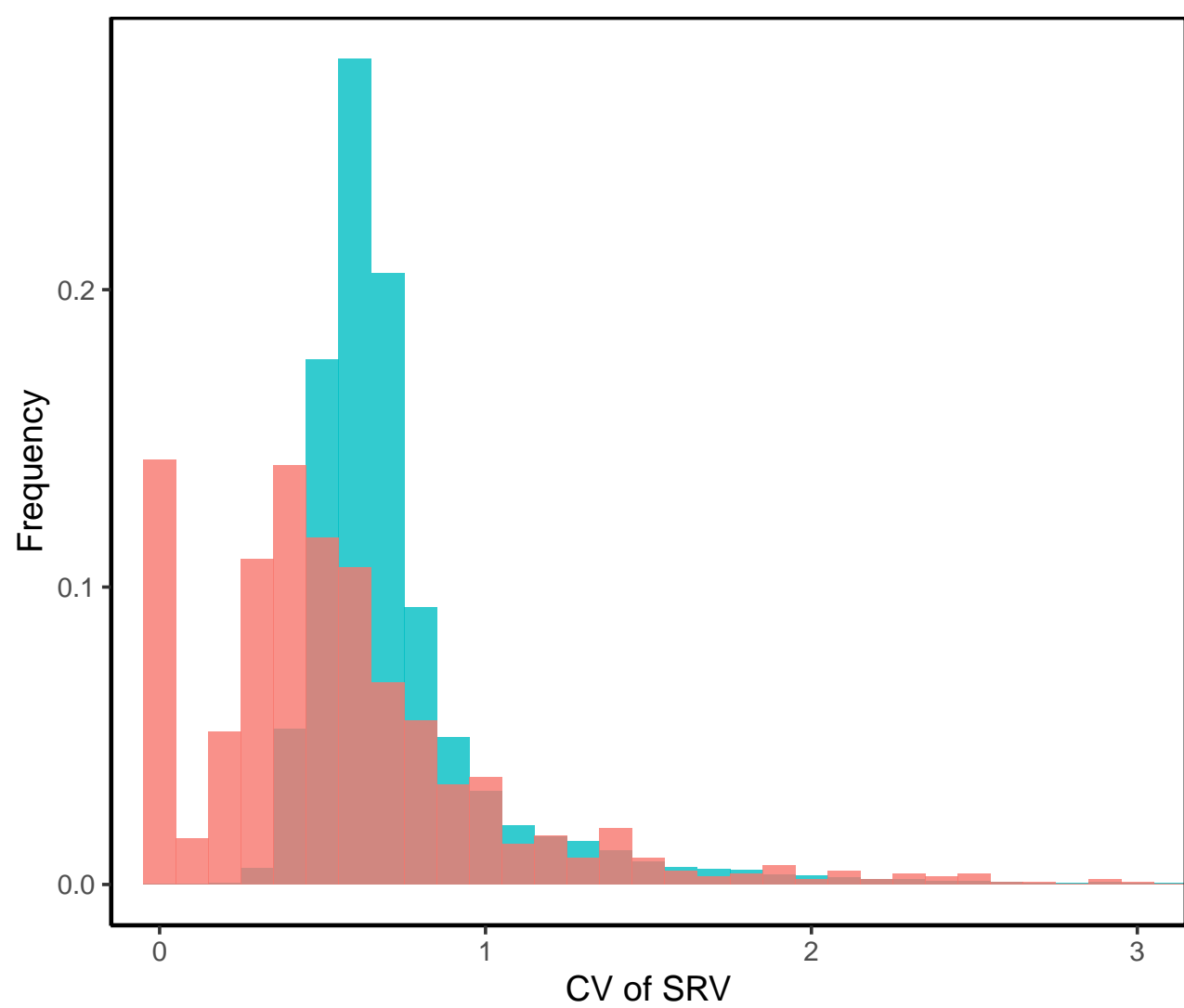

Supplement: msaa037_Supplementary_Data [file msaa037_supplementary_data.zip › msaa037-suppl-data/S2_CV_Hist.pdf]

## CV < 0.75

Count

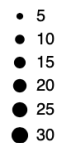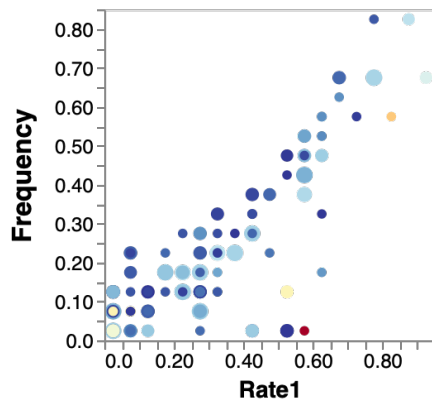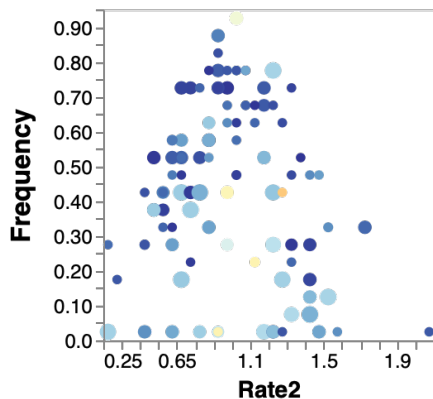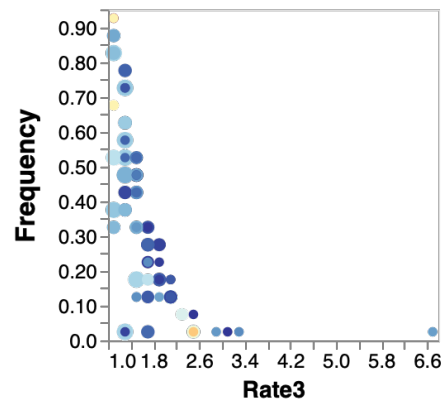

CV (a)

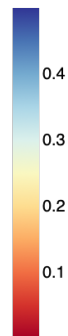

## 0.75 ≤ CV ≤ 1.25

Count

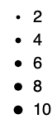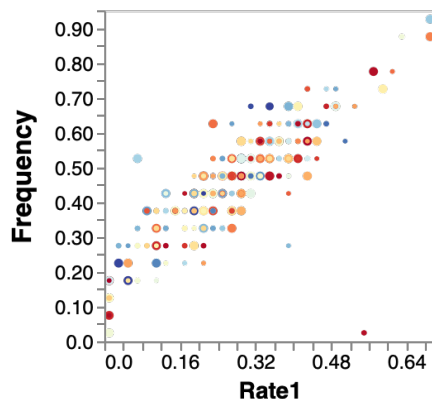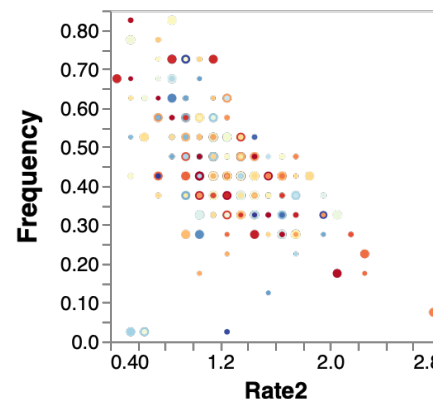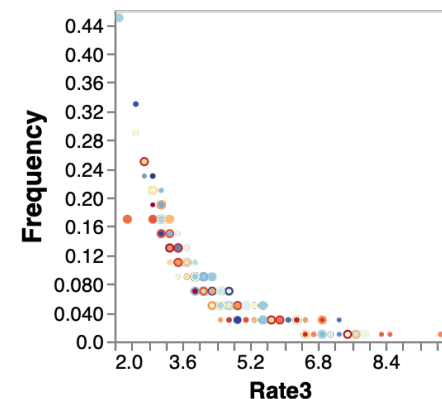

CV (a)

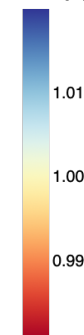

## CV ≥ 1.25

Count

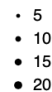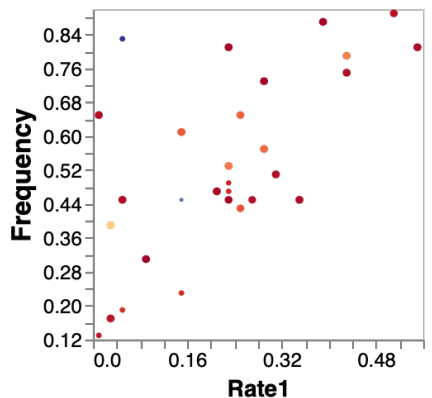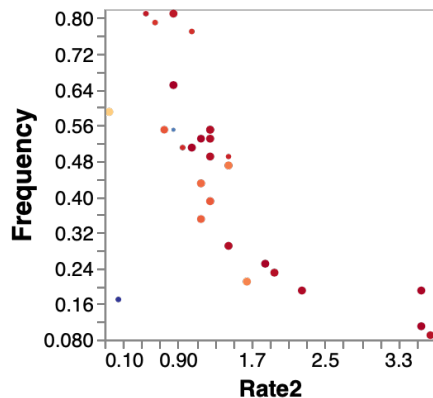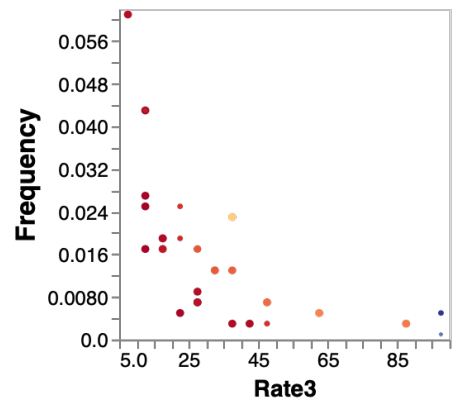

CV (a)

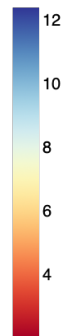

Supplement: msaa037_Supplementary_Data [file msaa037_supplementary_data.zip › msaa037-suppl-data/S3_Alpha_dist_FP_sims.pdf]

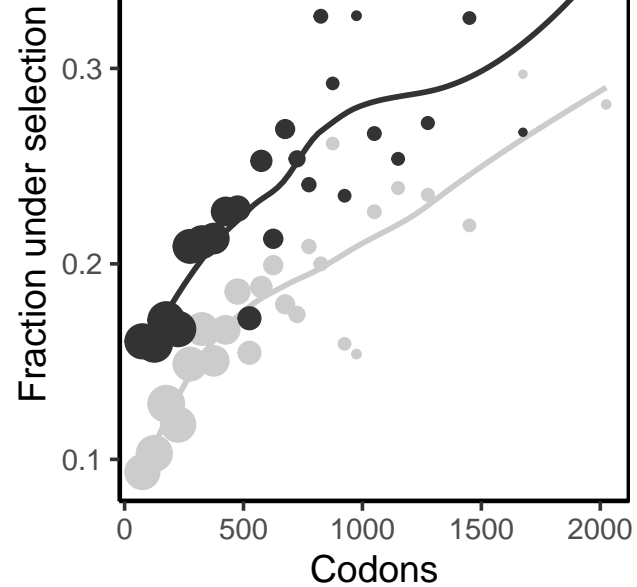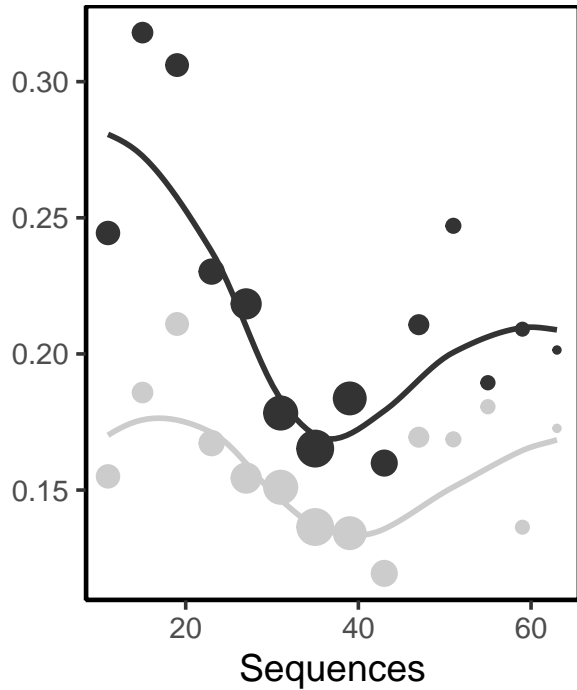

Supplement: msaa037_Supplementary_Data [file msaa037_supplementary_data.zip › msaa037-suppl-data/S4_Fraction_Under_Selection.pdf]

Sites

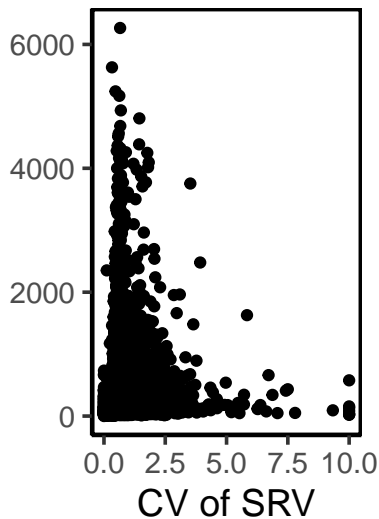

BUSTED  $\omega_3$

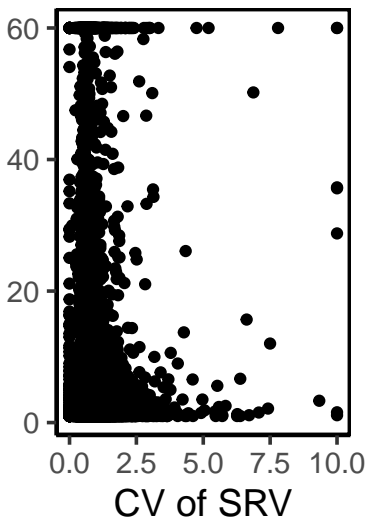

BUSTED Tree length

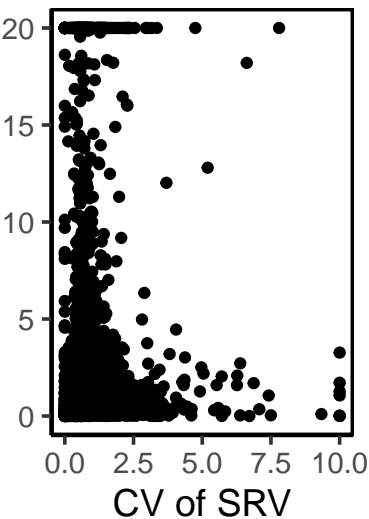

Sequences

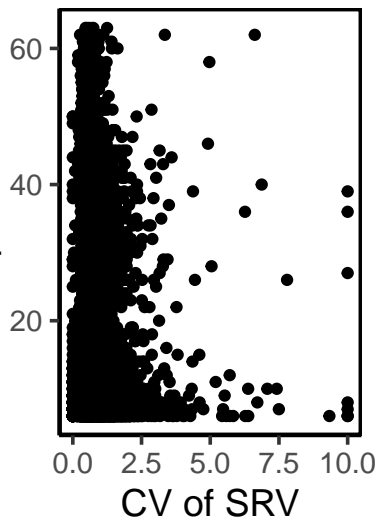

BUSTED[S]  $\omega_3$

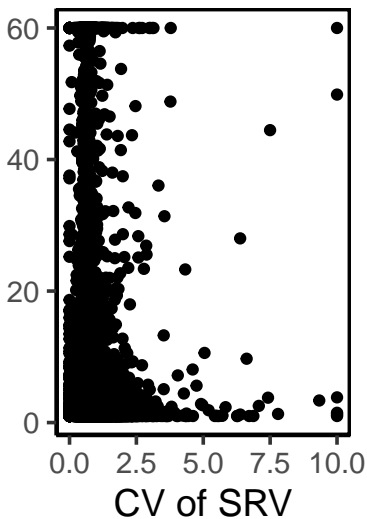

BUSTED[S] Tree length

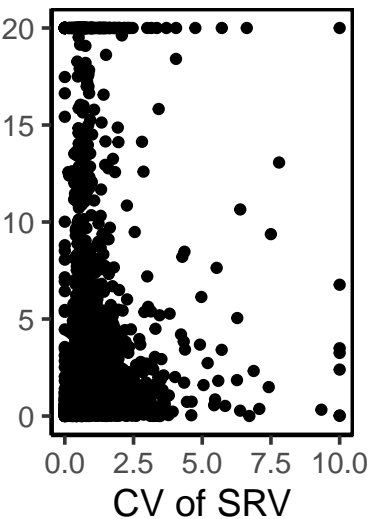

Supplement: msaa037_Supplementary_Data [file msaa037_supplementary_data.zip › msaa037-suppl-data/S5_CV_SRV_Relationships.pdf]

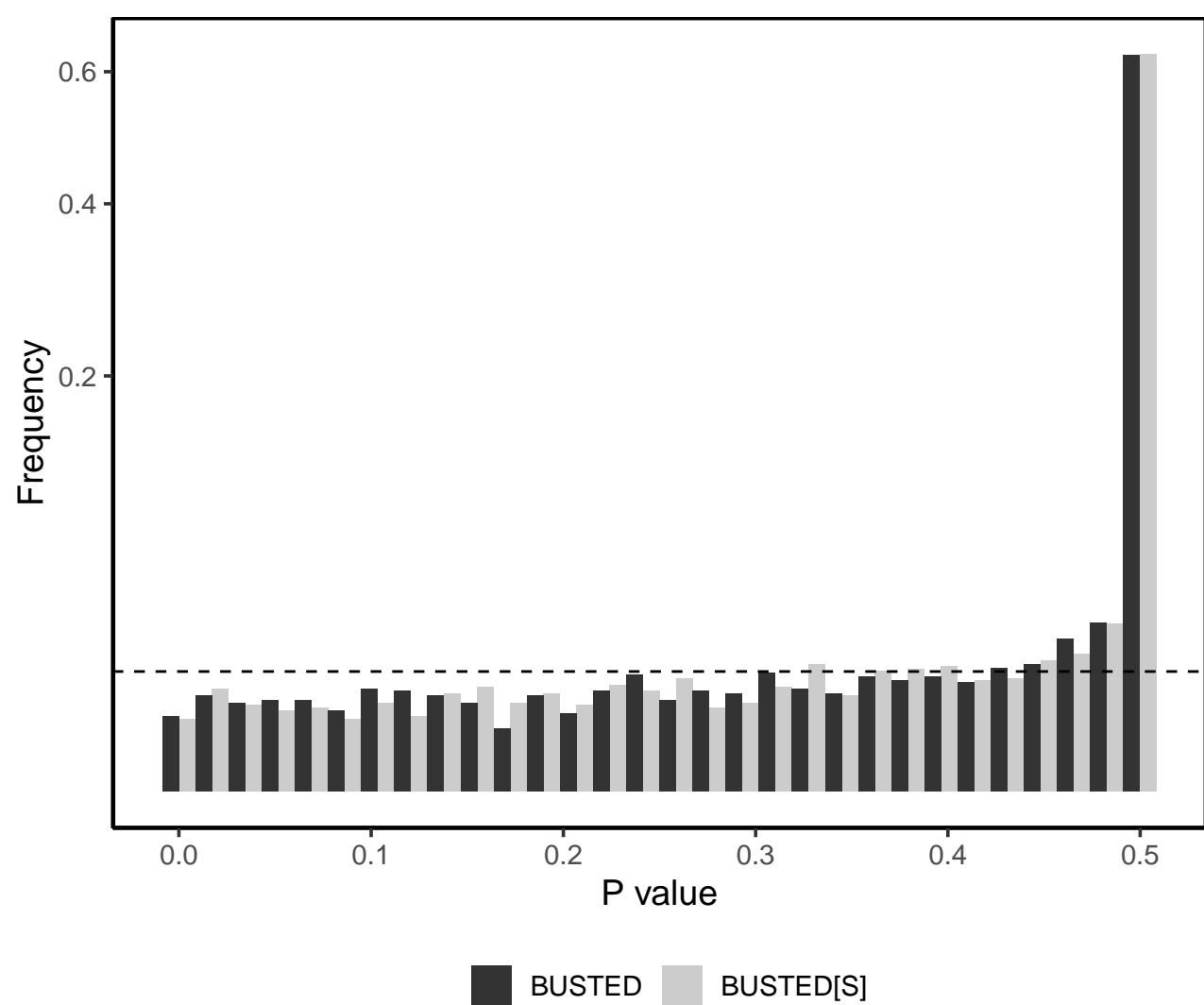

Supplement: msaa037_Supplementary_Data [file msaa037_supplementary_data.zip › msaa037-suppl-data/S6_P_Value_Distribution.pdf]

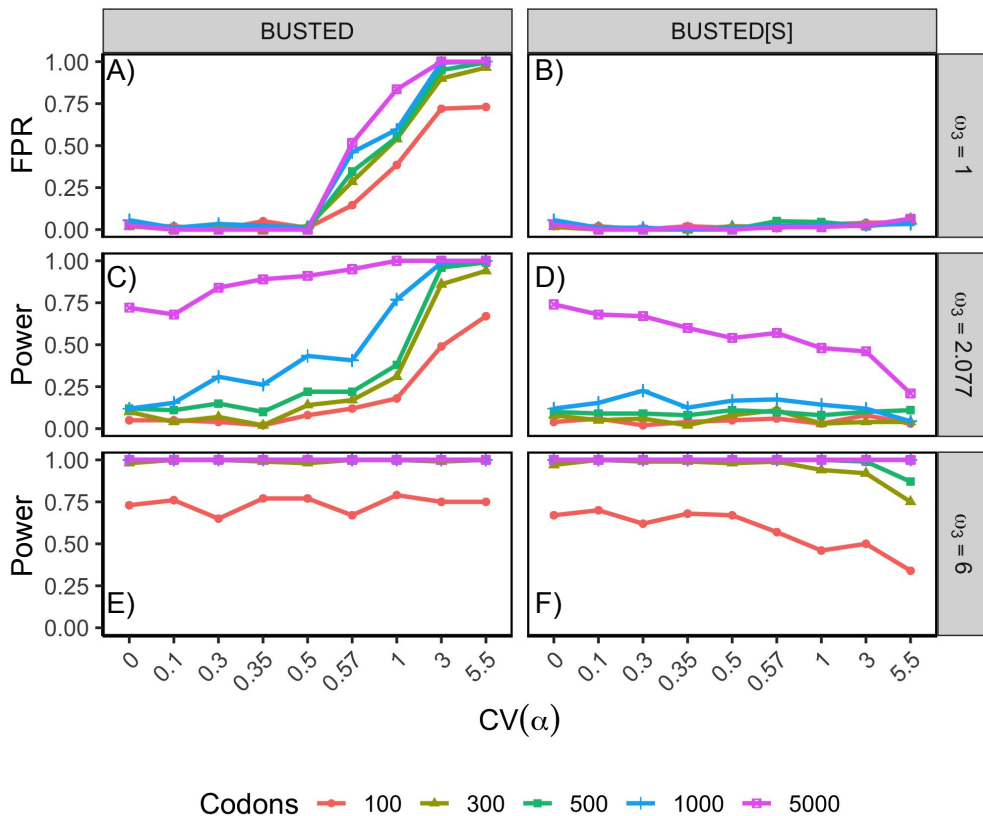

Supplement: msaa037_Supplementary_Data [file msaa037_supplementary_data.zip › msaa037-suppl-data/S7_Power_16_Seq_Sims.pdf]

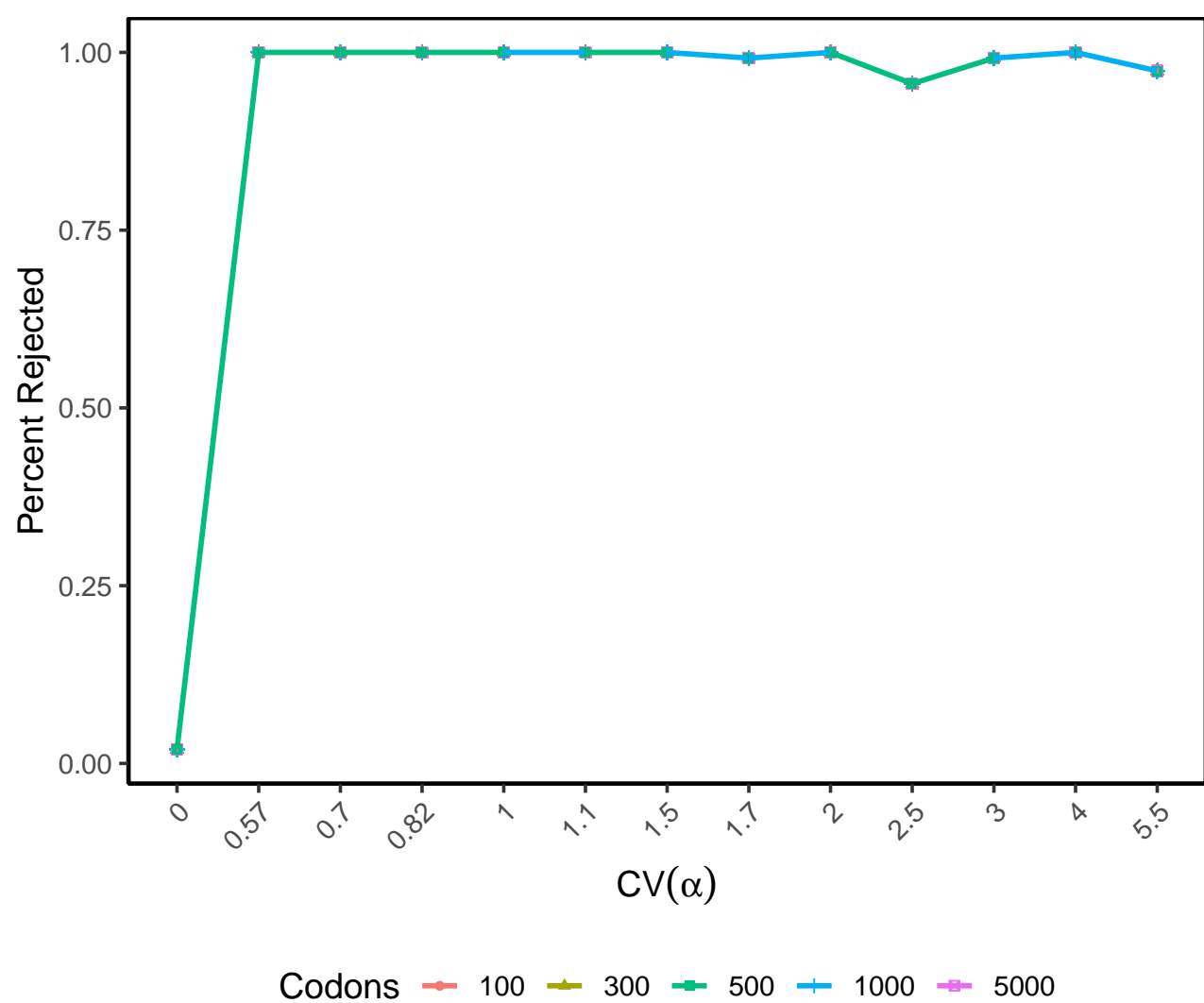

Supplement: msaa037_Supplementary_Data [file msaa037_supplementary_data.zip › msaa037-suppl-data/S8_False_Positive_PAML.pdf]

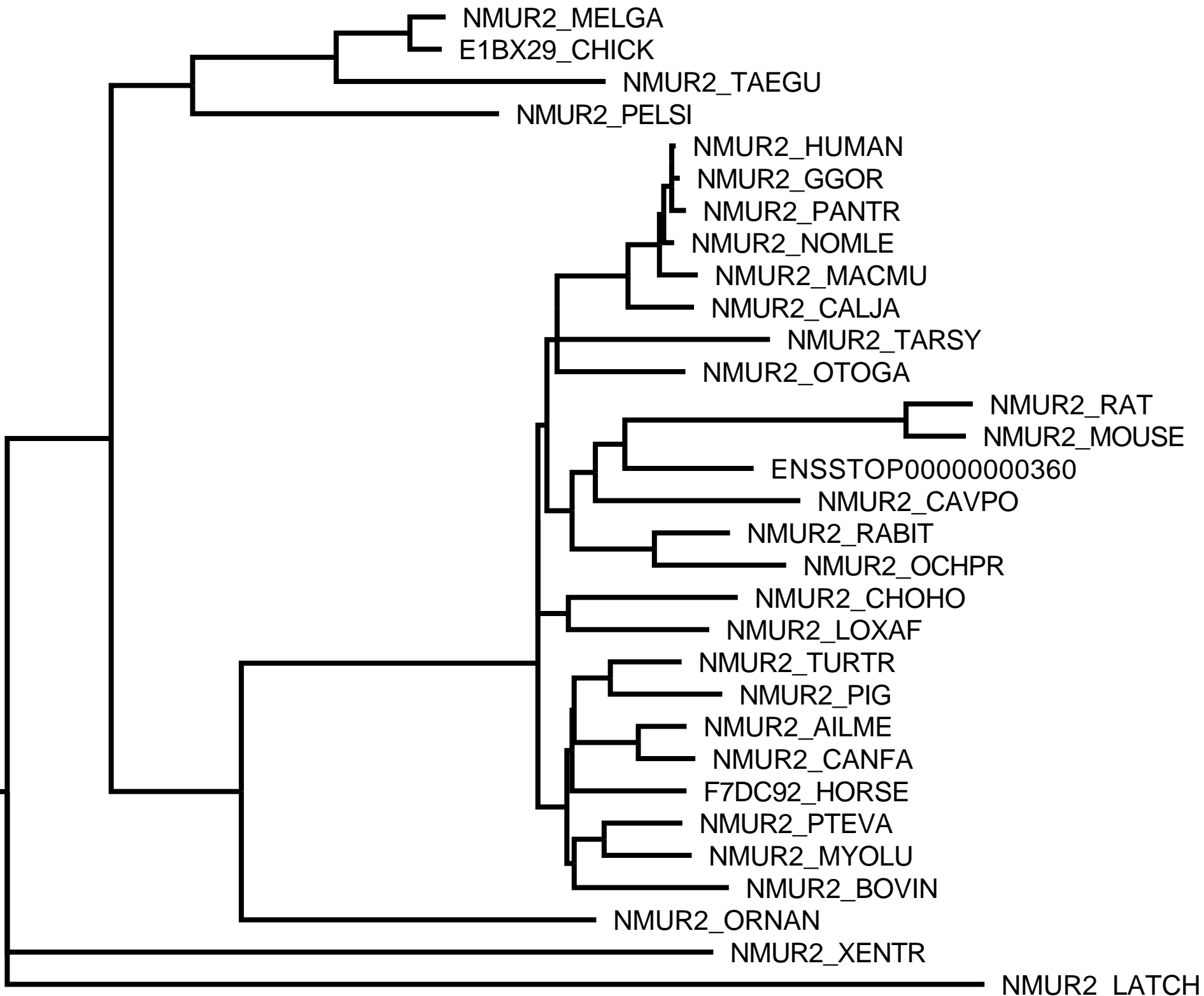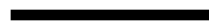

0.2

Supplement: msaa037_Supplementary_Data [file msaa037_supplementary_data.zip › msaa037-suppl-data/S9_Model_Tree_31_seq.pdf]
